# Hospital and Institutional News

**Published:** 1913-12-27

**Authors:** 


### HOSPITAL AND INSTITUTIONAL NEWS

### THE KING'S HOSPITAL PRESENTS.

The King and Queen, who were due to leave London on Monday for York Cottage, Sandringham, where Christmas and the New Year will be spent, have sent a number of Christmas presents to hospitals. In most cases the contributions of £5 have been accompanied by the stipulation that the money shall be spent on the entertainment of the patients at Christmas. Among the fortunate institutions are the Paddington Green Children's Hospital, of whose festivities we published some account a year ago, the Victoria Hospital for Sick Children, Chelsea, the Alexandra Hospital for Children with hip disease in Queen's Square, and the Cheyne Hospital for Sick and Incurable Children, and the Queen's Hospital for Children in Hackney Road. The "Evelina" has also received a gift, and the Queen has sent donations of £10 to the Royal Berkshire and Royal Portsmouth Hospitals, and a similar amount to the City of London Lying-in Hospital. Among the other provincial hospitals which receive gifts from the Queen we note the Royal Alexandra Hospital and Children's Convalescent Home, Rhyl.

### THE HIGH AVERAGE OF PROSPERITY.

There probably never was a time in the history of England when the average of prosperity of the business world in this country was so great as it has been during 1913. The newspapers are continually dinning in the fact of this prosperity, and anyone, who has had occasion to move about through the cities and towns, and in the highways and byways of Great Britain during the current year, must have found amazing evidence of the great prosperity of an immense number of people. Take the development of motor traction alone, and consider what that represents as a test of the prosperity existing amongst the middle classes, and even among the better paid of humbler classes too. Surely such evidence of the possession of ready money is remarkable. We made it our business to visit the United States before the McKinley Tariff was introduced, and in subsequent years. Before that tariff the possession of ready money amongst the masses of the workers was not sufficient to entitle it to be called "ready money." On our last visit in 1905, however, we travelled by railway, and came into contact with the American people in various parts of the States, and everything was changed. All the artisan classes and the majority of the workpeople everywhere, it soon became apparent, had money and ready money, too, in the real meaning of the word. Its possession made travelling and intercourse with the people infinitely more pleasant than it had been formerly, for it gave everyone a feeling of confidence, and removed from their shoulders the weight of care which small means and increasing expenditure produce in all ranks of life. To-day in Great Britain the bulk of the people ought to be cheerful, and they ought to shed the worn appearance which their faces often bear owing to the struggle for existence due to bad trade, or the absence of constant employment, with the suffering these always bring in their train. Judged by these tests the observer who studies the facial expressions of the classes we have here in mind, as he finds them almost everywhere throughout Great Britain to-day, may indeed thank God and take courage. As observers we have noted these encouraging facts, and, having faith in the people, we have turned with hope and confidence to the Business of Charity in an article on p. 328.

### HURRY UP INSTITUTIONS!

Now that Christmas is again with us, we hope once more to provide a full description of the decorations and entertainments which the happy ingenuity of institutional workers provides. The success of these is invariably a tribute to the inventiveness of individuals, and as new ideas in decorations, as in other matters, are sometimes hard to come by, it is of great value and amusement for one hospital or infirmary to know what another has achieved in this respect. We have often thought that many good ideas and beautiful effects are wasted through their reaching no larger public than that to which they primarily appeal, and therefore a record of the best that has been done each year provides a mine of information in our columns. Although many festivities extend to the New Year, the decorations, at least, are complete for Christmas. We therefore remind our readers that any ward of which the doers are particularly proud, any new entertainment or successful novelty, can be appreciated by other institutions if an account is sent to us not later than the first post on Monday, December 29. The stereotyped summaries sent out to the lay newspapers are useless, because the personal element of description and detail is lacking. When it is remembered that a notice last year on the present lines produced, among many delightful accounts, a description by the Chairman of Warrington Infirmary of possibly unique personal effort in the conduct of a festivities fund, we hope that confessions of similar interest will again reach us. In conclusion, preference will be given to those institutions of which accounts were not given last year. The cottage hospital, the sanatorium have each something unique to tell, and we hope they will be prompt to share it.

### THE DINNER TO M. ANATOLE FRANCE.

Among medical men resident in institutions is a certain proportion, not large but noticeable enough, who have eschewed the cares of general practice in order to have time for some intellectual hobby, often connected with literature. If one's acquaintance is large enough, bibliophiles or Egyptologists, or black-letter text enthusiasts may easily be met with. It is to such as these that we address the remark that it is surprising to note among the names of those to be present at the complimentary dinner to the great French writer none that belong to medical men. We fancy, too, that we know the reason of this. To justify its existence in space (in the esoteric sense) to an editor, a name must have special eminence. Now our eminent medical men, nearly every one, have enough to do of their own professional work without indulging in *parerga*, or rather, to avoid magniloquence, let us say in *Nebenberufen*. The true taster and appreciator of literary excellence may have no excellence of his own, in a professional regard. There is a gentleman of this kind, known only to a small circle—successful professionally, too, we are glad to say, in a modest way—who, long ago before M. France became well known and fashionable on this side of the channel, would recount to his friends the delight he took in making a translation of *Thais*. This is the unoffending sort of person whom Mr. Stephen Paget is so hard upon. But to stick to the shop-board, except for regular visits to church, is not, as we have hinted above, the ideal of quite every medical man.

### FIRST COMPLAINT AGAINST THE NEW "KING'S."

At Southwark Coroner's Court allegations of sending away a patient on the part of the porter of King's College Hospital were recently made. It appeared from the evidence that as the result of a rash a mother took her son aged five years to the hospital, where she told the porter that he was suffering from a skin complaint. He replied, she said, that she must come again in two days' time, when the dermatologist would be in attendance. On her saying that the child was too ill to wait a clerk told her to go to the casual ward. As the child was worse next day she took him to the Evelina Hospital, where he died shortly after admission. In reply to the Coroner the mother said she was not satisfied, and asked why she and her sick child should be turned away. F. C. Acteson, the hospital porter, said that he saw 200 patients daily, and did not remember the previous witness, but that it would be a very serious thing for him to send a patient away. The mother must have been mistaken. Dr. Ashe, the senior resident casualty officer at King's College Hospital, said that, if the mother had told the porter that the child was ill, he would have sent them at once to the out-patient department. The mother had been taken to the hospital by the Coroner's officer, who said that she had identified the porter. Recalled, she repeated her previous statement, and the jury returned a verdict of death from natural causes, adding that they were of opinion that the porter should have been a little more careful. Dr. Ashe said that it would be a serious thing for the porter if the mother were proved correct, and that the hospital's authorities would inquire into the matter. The subject is, therefore, not ripe for comment. We can so merely record what is probably the first complaint preferred against the new King's College Hospital.

### THE CONDITION OF CARDIFF WORKHOUSE.

Mr. H. R. Williams, the Local Government Board Inspector, has laid an unanswerable case before the Cardiff Guardians for the separation of the infirmary from the workhouse. At a recent meeting he described the condition of the workhouse as most serious. It had become, he added, almost wholly an infirmary, and he reminded the Board that it was so far back as two years ago that he had advised them to build a separate institution. Mr. Williams then pointed out the drawbacks attaching to the presence of a number of mixed cases of serious disease under one roof, and to the absence of a separate ward for children. "You are behind the times," he is reported to have said, and indeed, these defects are glaring. Archdeacon Buckley presided on this occasion, and no doubt realised that such a comment has a real sting at Cardiff, where King Edward VII Hospital has set such a well-known example of progressive development, and where the Cardiff Mental Hospital is equally recognised as in the van of progress. We can hardly believe that the Cardiff Guardians will be content to brand themselves as men of a different calibre from those who form the boards of the voluntary hospital and the mental hospital. Nor surely will the public, who have supported and endorsed the two institutions mentioned, allow the public infirmary of the city to remain behind the times. That is an anomaly unworthy of the capital of Wales.

### THE NEW BURY INFIRMARY.

Alderman Mitchell, the deputy-mayor of Bury St. Edmunds, opened last week the new Union Infirmary, which has been built on account of the overcrowding. Connected with the old building by a covered way, the extension, which is for the male side, consists of a main ward for 18 beds, with a separate sanitary block, 4 nurses' rooms, a dayroom, and accommodation for linen and stores. The first floor is similarly planned, except that it contains the quarters of the resident medical officer, Dr. H. G. Kilner. The ward floors are of pitch-pine with a Ronuk finish, and the architect is Mr. Sidney Naish, of Bury. The south frontage of the extension is furnished with a verandah and balcony, partially protected from the weather. The extension has been carried out for £3,600, a sum which includes the cost of furnishing and equipment, and Mr. O. D. Jonson expressed his opinion at the opening that it contains ample provision for their requirements. We hope that this is the fact, for there has been such a levelling-up of infirmaries of late years that the former pernicious pride in their parsimony and forlornness has been replaced by a generous rivalry determined to make them as efficient as any class of institution for the sick.

### THE COMING POOR-LAW CONFERENCE.

On Tuesday and Wednesday, February 17 and 18, the Central Poor-Law Conference will meet, by permission of the Corporation, in the Council Chamber of the Guildhall, when the Lord Mayor will open the proceedings. It is announced that Lord Methuen will preside, and the delegates include Lord Richard Cavendish, who is chairman of the Central Committee of Poor-Law Conferences, representing the Ulverston Board.

### EASTES v. RUSS: RESULT OF APPEAL.

The Court of Appeal has dismissed an appeal from the decision of Mr. Justice Sargant, which was recorded and discussed in The Hospital of May 31 and June 7 last, in which he held that the defendant, a pathologist and former assistant of the plaintiff, was prevented only during the period of his engagement, from practising within ten miles of the former's laboratories. In dismissing the appeal the Master of the Rolls said that the real difficulty was whether the restraint, which he thought, in opposition to Mr. Justice Sargant, was imposed for the whole of the defendant's life, was reasonable or not. He held that to exclude all competition was neither necessary nor reasonable since the public interests had to be considered. Lord Justice Phillimore concurred, but Lord Justice Swinfen Eady, in a dissenting judgment, said that the defendant possessed a very extensive field of work outside the Harley Street area, and that the limitation for the whole of his life was not longer than was reasonable. As we said before in commenting on Mr. Justice Sargant's judgment, whether reasonable or not such a limitation is very drastic—so drastic, indeed that, if fully aware of it, it seems to us doubtful if any one would so bind himself, when engaging to work with Dr. Eastes.

### UNQUALIFIED PRACTITIONERS AND THEIR FORTUNES.

Those who share the common interest in the fortunes acquired by men once qualified whose personal energies have been able to surmount the condemnation of the General Medical Council will read with interest that "Dr." Edward William Alabone has left estate of which £25,041 is net personalty. The well-known "consumption specialist" had obviously made a good thing out of his treatment, but the above announcement throws no light on his possession or otherwise of real property. The truth, of course, is that any man who has capacity for surviving official excommunication from his profession must be at any rate a good man of business, whatever else he may be, and as such likely to succeed financially in any undertaking. Still, it is not every qualified practitioner whose net personalty runs into five figures, and as a matter of history Mr. Alabone's estate deserves record.

### TUBERCULOSIS DISPENSARIES FOR LONDON.

In a circular issued by the Local Government Board the Metropolitan borough councils are invited to provide dispensaries to be co-ordinated with the County Council, which will help to systematise all the agencies at work. A meeting of the twelve general hospitals with medical schools recommended, and the Board endorses the view, that all dispensaries should use these hospitals for consultative purposes, and that they should, where they desired, act as tuberculosis dispensaries, Where dispensaries are non-existent or inadequate for the work, they should be established by the borough councils singly or in combination. The Board holds that beds should be available for observation cases, and that these should not be provided at the dispensary, but by the County Council. The circular co-ordinates the dispensary work with the preventive work of the borough under the Tuberculosis Regulations, 1912. Financially, the borough councils are to be regarded as the providing authorities for dispensaries which will serve the whole community, including insured persons, and should contribute lump sums in respect of the whole borough rather than fees per case to voluntary dispensaries. The Insurance Committee should pay the borough council for the dispensary treatment of insured persons, after deducting which their expenditure will be met by 50 per cent. from the Board, 25 per cent. from the London County Council, and 25 per cent. out of the rates. The Board states that the London County Council are prepared to provide or arrange residential accommodation if the cost of treating insured persons is borne by the Insurance Committee.

### HOSPITAL PROFITS BY INDUSTRIAL DISPUTE.

An unusual clause in the agreement arrived at recently between the Great Western Railway Company and their employees who had taken part in a "sympathetic strike" was that providing for donations of one shilling to half-a-crown by each workman to the funds of the Swindon Victoria Hospital. This railway town is the most important centre on the Great Western system for construction and repairing workshops and similar railway manufactures, so that at first sight it might appear as if the contributions would directly benefit the employees making them. This, however, is not strictly the case, for there is at Swindon a hospital of fifteen beds supported entirely by the railwaymen and by the company. The Victoria Hospital has, according to "Burdett's Hospitals and Charities," twenty-four beds and five cots. It receives, doubtless, principally the dependants of the railwaymen, so that indirectly the latter stand to benefit by its prosperity. The annual ordinary income is stated as £820; a very modest sum considering that 408 in-patients were received and over 1,500 visits paid to out-patients. Inasmuch as something over 2,000 railwaymen were involved in the strike, the hospital stands to benefit to the tune of between £100 and £200, no small proportion of the total ordinary expenditure. It is an ill wind that blows nobody any good; but it is not often that a strike benefits a voluntary hospital. It will be remembered that £11,000 is wanted for capital expenditure at this hospital, as was recorded in our "Buildings Contemplated" of December 13.

### FINANCIAL MISMANAGEMENT AT DEVONPORT.

A special committee of governors has been held to consider the financial position of the Royal Albert Hospital, Devonport, the ordinary receipts of which last year fell from £3,487 to £2,633, while there was a deficit on the year of £1,736. Mr. Lewis Foster, the treasurer, who explained the position, said that the wards were always full, and that if the hospital was going to be carried on as at present £4,500 a year was really required. There was an annual decrease in subscriptions, and donations did not grow in numbers. Mr. W. J. Waycott urged that the support of the Mayor should be invoked, seeing that working classes did not do more to support the institution by which so much benefit was conferred upon them. Mr. F. J. Bone said that he believed the position to be more serious than any of them realised, and he was afraid that the result of a public meeting would not be that they would have sufficient support to carry on the hospital much longer. It seemed to him that the institution must either be handed over to the Corporation or else closed. A special committee was appointed to consider how best to raise funds and to report. By a wise provision it was decided that not governors only but also "influential members of the general public" should be placed upon this special committee. We cannot believe that a town like Devonport, with its traditions of personal service and with the lively feeling of responsibilty and public spirit which may be expected in such a centre of the naval and military services, for a moment will permit the present slur to remain upon its inhabitants. A town which has hitherto supported its hospital for fifty-two years, which still fills its hospital beds with the utmost regularity, and which has increased its own prosperity during the meantime, is not going to be the only community in the country to pretend that the voluntary system has broken down. One speaker, Mr. Sanders, actually affirmed that he did not know more than one or two big establishments which kept a box and paid so much a week to the hospital; two others said that employers were doing little, and that eight or nine who previously subscribed to Hospital Saturday had sent nothing at all this year. It should not take much cogitation upon the part of the special committee to see that, the financial crisis which is overshadowing the Royal Albert Hospital has been occasioned, by letting the ordinary means of stimulating interest and organising annual subscriptions fall into disuse. Get a weekly box in every big establishment and the battle is half over. The cry for State aid is the cry of want of initiative.

### CHRISTMAS ADVERTISING.

One of the questions asked in the "Institutional Worker Supplement" for this month was the following: "How can the opportunities afforded during the Christmas season best be made use of for advertising the hospital and increasing its income?" It is a question that many London hospital officers might well feel hesitation in answering, for surely one of the most depressing sights in the world at this time is the column or more headed "Hospital Appeals" appearing in one page or another of the daily newspapers. The process, we regret to say, seems to be for some secretaries, to relegate to one of their subordinates a perfunctory paragraph of a few hundred words without a spark of individuality or original interest, to have this solemnly reproduced on flimsy paper, and sent out as a matter of routine to all the papers. The result is the deadliest of dull columns—a masterpiece of fatuity. The practice would probably be defended on the ground that editors are overwhelmed with "copy" at this season, and will not look at an appeal if it is not short. The Christmas festivities in hospitals are perfunctorily described in the same way. We remember last year noticing that the accounts, with the rarest exceptions, were so colourless that, the names of the institutions concerned could have been changed in nine cases out of ten without the smallest sense of incongruity. It would be much better for a hospital to approach one or two papers beforehand, to suggest a really carefully written intimate individual account, than to be satisfied with the present general paragraph, the only use of which is the mention of the institution's name. Such descriptions will be printed, if they are made interesting—if, in a word, trouble is taken with them—as the accounts published in The Hospital of last year remain to prove. Undoubtedly the provinces can teach London institutions much in respect to Christmas advertising, for in the provinces the appeals and descriptions of the Christmas season are the personal pride of the man, who writes them.

### MARGARINE IN INFIRMARIES.

The Local Government Board have issued a Memorandum to the Bermondsey Board of Guardians, in which they dilate on the calorific value of margarine as compared to butter. They state that various investigations have been undertaken to ascertain the comparative nutritive values of margarine and butter, and the general conclusion arrived at is that there is no appreciable difference. The Memorandum concludes by stating that "the advantage which butter possesses over margarine is æsthetic rather than dietetic, and the difference in price between these two articles represents what the consumer is willing to pay for luxury." The Bermondsey Guardians decided to substitute butter for margarine in their infirmary some weeks ago at an extra cost of £1,500 a year, but on receiving the foregoing Memorandum they have now decided to revert to the use of margarine.

### RESEARCH WORK AT THE BROMPTON HOSPITAL.

Lord Cheylesmore, chairman of the committee of management of the Brompton Hospital, has received a letter from the Maharajah of Nepal, announcing that an anonymous donor is ready to provide £1,300 for the endowment of research at the institution. The work which the hospital has accomplished in this respect, thanks largely to the Maharajah's gift two years ago, is already of interest, but to continue it, Lord Cheylesmore points out, trained workers are necessary, and Dr. A. C. Inman with his staff and laboratory provide a nucleus which more money is needed to extend. A further £5,000 is asked for, and should be forthcoming if the benevolent public will only bethink themselves of the necessity for prosecuting research at a time when so much public money is being spent on treatment. Research, adequately prosecuted and with public support behind it, is more needed now than ever, so that the anti-phthisis efforts now in being may be guided by scientific knowledge of the best means to prevent the disease by checking it in early cases and by preventing any new causes of infection which may be revealed. It has long been hoped by scientists to discover a practical test of the presence of tubercle, such as that which the Wassermann reaction provides for syphilis, a hope, surely, which might well inspire married men with imagination to subscribe towards this end.

### ANTI-PHTHISIS CONVALESCENT HOMES.

Another aspect of child welfare may be seen in connection with the Samuel Lewis Convalescent Home at Walton-on-the-Naze. The Home controls a cottage which accommodates a number of children drawn from those homes in the East End of London where there is a consumptive parent or other member of the family, or where the conditions are a menace to the child's future health; where the child is perhaps on the verge of consumption. These are cases in which a year or two in the country perhaps may make the deciding point in its life. The children, we understand, attend the local school and are cared for by a housekeeper under the supervision of the matron at the Convalescent Home, who visits regularly. The Home doctor also visits and inspects the children once a week and whenever wanted. The success of the plan may be judged by the fact that an increase is contemplated.

### A NEW INFIRMARY FOR LAMBETH?

It is evident from the latest returns of the Poor-Law infirmaries of South London that there will be no diminution in the number of patients owing to the National Insurance Act. At Lambeth it was anticipated by the chairman, Mr. Frank Briant, L.C.C., who is a member of the London Insurance Committee, that their numbers would fall off, and in view of the general anticipation it was decided by the Guardians, twelve months ago, to adjourn consideration of any scheme for the erection of a new infirmary. Whilst they have come to the conclusion that it would be inadvisable to erect a new infirmary, the board has decided to increase the accommodation at the present building. The classification of the inmates at the workhouses is to be reconsidered with a view to removing from the infirmary all chronic cases of illness, and by this means to relieve the existing pressure at that institution. Considering all the circumstances, it is doubtful whether it would not be more advantageous and economical for the Guardians to revert to their original proposal—the erection of a new infirmary.

### THE PUNISHMENT OF PANEL DOCTORS.

A panel doctor has been complained of before the Insurance Committee of the County of London as behaving in an offensive and familiar manner towards an insured person. The report of the Medical Service Sub-Committee was to the effect that the practitioner had behaved in a "most offensive and unprofessional manner," and their recommendation was that he be severely censured, and that his patient be transferred to another doctor. Mr. Kingsley Wood thereupon inquired how the censure would punish the panel doctor, whereupon Mr. Warbrer, the Chairman of the Medical Service Sub-Committee, said that only two alternatives were open to the Committee—to censure, or to report the case either to the Insurance Commissioners or to the General Medical Council. No doubt the latter course would be a sufficiently drastic procedure—too drastic, indeed, for all but the most serious breaches of professional conduct. The vote of censure on the part of a local Insurance Committee may not mean much in itself, but were it included in the minutes of the committee's proceedings, which are made public, such a vote might be a severe blow to the practice of the offending practitioner. We are inclined to think, indeed, that offensive treatment on the part of a doctor to a patient is so soon spread and bruited abroad that his list will shrink considerably. The present case should be useful in impressing on panel doctors and patients alike that, whatever be the disadvantages of the system, professional behaviour is as strictly insisted on as elsewhere. It is to be noted that contract practice in all forms is always more prolific of insult to both doctors and patients than the more personal relation of private practice.

### THE BEIT RESEARCH FELLOWSHIPS.

The fifth annual election to the Beit Memorial Fellowship was made at a meeting of the trustees last week. This year eleven elections were made, and once more three of the Fellowships are given to women, who therefore cannot feel aggrieved that a due proportion is not allocated to their sex. One is a graduate of Sydney and one of Toronto. Four propose to undertake their researches at the Lister Institute, and are, we note, at the Cambridge Research Hospital. In one instance—namely that of Miss Annie Porter, a graduate of London University—the place of research is multiple: the Quick Laboratory at Cambridge, the Liverpool School of Tropical Medicine, and, if possible, the King Institute of Preventive Medicine, Madras, and the Wellcome Research Laboratories at Khartoum are being mentioned. We remarked last year that only one of the Fellows proposed to undertake research work abroad, and the list again shows that the Fellowships are being granted in a cosmopolitan spirit. It will be remembered that each Fellowship, which is worth £250 a year, is granted for three years, but that the trustees have a power, which does not seem to have been exercised on this occasion, to extend its terms for a further year. There were thirty-five candidates, and one more election than was the case a year ago.

### THE NEW YEAR'S REFERENCE BOOKS.

The old prejudice against reference books as merely a form of advertising is dying before the need in modern civilisation of securing information promptly concerning every movement, and those who are responsible for any part in it. No one, perhaps, is more in need of such information than the hospital secretary, who, if he is worthy of his post, touches life at innumerable points, and must himself be a sort of walking reference book at committees. The increasing number of this class of book is therefore on the whole to be welcomed. Apart from "Burdett's Hospitals and Charities," which is the mainstay of information for institutional workers, "Who's Who" is an essential from the all-embracingness of its scope. The companion volume, also published by Messrs. Black, for one shilling, is the "Who's Who Year-Book," which, as the publishers justly point out, forms the reverse reference. It is an admirable directory of positions and classes of posts, without which the directly biographical volume is incomplete. Publicity is now such an important subdivision of secretarial work that A. and C. Black's "Writers' and Artists' Year-Book" is most useful for reference to publications, even for those who, like secretaries, are not often free-lances themselves. One of the most striking enlargements of a book of reference is the "Medical Who's Who," published by the London and County Press Association, the 1914 edition of which is a much fatter volume than its predecessors. Now that its inclusiveness and the absence of selection possibly implied by its title are becoming recognised, it is making headway, and the clearness of its information makes it more easy to handle than some of the larger books of its class. Another useful publication is the "Englishwoman's Year-Book and Directory," published by Black, price half-a-crown, which, in view of the increasing part which women are taking in public and social matters and charitable work, contains answers to many of the questions likely to be asked before the year is over. The reference book is often more satisfactory than the telephone. One can reach the source of information more directly, and one of the tests of the up-to-date secretary is the care that he takes over his equipment in this respect.

### MEDICAL TRAVELLING PAST AND PRESENT.

The accidental death of a famous racing motorist, M. Jenatzy, which took place recently in Belgium, reminds one how very greatly facilities of visiting patients have improved in the last decade. It is only ten years since the person named won the then famous Gordon-Bennett race in Ireland. At that time the percentage of medical men using automobiles for their professional work was negligible. Automobilism was a perilous sport, confined to heroes with a strong infusion of the engineer, somewhat as aviation is nowadays. And yet many who then looked with awe on those who sat behind a steering wheel had it in them to excel in like manner. M. Jenatzy—who achieved, by the way, that mark of supreme excellence in sport, namely, having a racehorse named after him—was physically of the type of many of our younger surgeons; a small man with extremely strong, rough-hewn, well-defined features, small eyed, formidable, even sinister, looking. The Irish crowd—the writer well remembers seeing him race—dubbed him "Satan." It was a confirmation of the Goethian conception of identifying ability with evil; a conception also to be traced in Chaucer's Leech and Balzac's Despleins. But the doctor nowadays, whether he "scorches" or not, is, in the main, of highly respectable behaviour. And undoubtedly for the fact that, if not too much panelised, he is less of a peripatetic drudge than he used to be he has the automobile and nothing else to thank. The saving of time has been a godsend to many practitioners getting up in years.

### GRANTS TO LONDON TUBERCULOSIS DISPENSARIES.

At a meeting of the London Insurance Committee last week the question of grants to Metropolitan boroughs, in respect of dispensary treatment of insured persons suffering from tuberculosis, was discussed. Mr. Edward Smith, the Chairman of the Finance Sub-Committee, objected to a proposal that a grant of £100 be given to Wandsworth on the ground that St. Pancras, under the same head, received a grant of only 7s. 5d. per head per annum; Deptford, £1; while the proposed grant would work out at 16s. 8d. He pleaded for fair treatment to all the boroughs. Payment by capitation was a possibility to be remembered. It was then insisted by Mr. Warburg that to be eligible for grants tuberculosis dispensaries must be affiliated to a hospital, in order that difficult cases might be adequately treated. Mr. S. A. Dawes, the Chairman, then explained the variability of the conditions in different districts, and the grant was agreed to. We have recently pointed out the desirability of having dispensaries connected with hospitals, while showing also how much they differ, or should differ, from the ordinary out-patient department. It is important that if the £5,000 is to be spent to the most advantage, some recognisable principle should be made clear as to the considerations which weigh most with the Committee if a sense of injustice is not to arise among the different boroughs.

### DOCTOR'S BEQUEST TO ST. BARTHOLOMEW'S HOSPITAL.

A splendid bequest to St. Bartholomew's Hospital is announced, which will relieve some, if not the whole, of the more pressing necessities of that venerable institution, lately in the unaccustomed situation of having to seek for aid beyond its own endowed revenues. Mr. John Moore Walker, M.B., of Edgbaston, has left the residue of his property to St. Bartholomew's, his old hospital when a medical student; and this will benefit the institution to the extent of about £30,000. Mr. Walker, although a Bart.'s man, took his qualifications in Edinburgh some forty-two years ago. It is evident that he was very loyal to his old hospital; and if controversies should ever arise—which heaven forfend—as to the relative benefits received from each other by the hospital and school respectively, the school might claim to have this magnificent legacy placed to its own credit in striking a balance with the hospital!

